# Redefining Knee Balance in a Medially Stabilized Prosthesis: An *In-Vitro* Study

**DOI:** 10.2174/1874325001711011165

**Published:** 2017-10-31

**Authors:** Philippe Van Overschelde, Vera Pinskerova, Peter P. Koch, Christophe Fornasieri, Sandro Fucentese

**Affiliations:** 1 AZ Maria Middelares, Buitenring Sint-Denijs 30, 9000 Gent , Belgium; 2 Charles University, First Orthopaedic Clinic, Faculty of Medicine, Prague, Czech Republic; 3 Kantonsspital Winterthur, Klinik für Orthopädie und Traumatologie, Brauerstrasse 15, 8400 Winterthur, Switzerland; 4 Clinique Générale Annecy, 4 Chemin de la Tour la Reine, 74000 Annecy, France; 5 University Hospital Balgrist, Forchstrasse 340, 8008 Zürich, Switzerland

**Keywords:** Total knee arthroplasty, Prosthetic design, Cadaveric study, Ligament balancing, Medially stabilized knee, Ligament release

## Abstract

**Background:**

To date, there is still no consensus on what soft tissues must be preserved and what structures can be safely released during total knee arthroplasty (TKA) with a medially stabilized implant.

**Objective:**

The aim of this study was to analyze the effect of a progressive selective release of the medial and lateral soft tissues in a knee implanted with a medially stabilized prosthesis.

**Method:**

Six cadaveric fresh-frozen full leg specimens were tested. In each case, kinematic pattern and mediolateral laxity were measured in three stages: firstly, prior to implantation; secondly, after the implantation of the trial components, but before any soft tissue release; and thirdly, progressively as soft tissue was released with the trial implant in place. The incremental impact of each selective release on knee balance was then analyzed.

**Results:**

In all cases sagittal stability was not affected by the progressive release of the lateral soft tissue envelope. It was possible to perform progressive lateral release provided the anterior one-third of the iliotibial band (ITB) remained intact. Progressive medial release could be performed on the medial side provided the anterior fibers of the superficial medial collateral ligament (sMCL) remained intact.

**Conclusion:**

The medially conforming implant remains stable provided the anterior fibers of sMCL and the anterior fibers of the ITB remain intact. The implant’s sagittal stability is mainly dependent on its medial ball-in-socket design.

## 1 INTRODUCTION

In the native knee, during flexion, the medial contact point remains almost fixed, whereas the lateral contact point of the femur translates posteriorly [1]. The different kinematics of the medial and lateral compartments cause the tibia to rotate internally and the lateral femoral condyle to translate posteriorly during flexion. However, current total knee arthroplasty (TKA) designs (cruciate retaining, posterior stabilized, and mobile-bearing) do not provide normal kinematics, and “paradoxical” anterior translation of the femur on the tibia during flexion is common [2]. Paradoxical anterior femoral translation has been reported to result in a more anterior flexion axis, leading to a lower range of flexion, a lower quadriceps efficiency due to decreased quadriceps moment arm, and increased polyethylene wear [2, 3].

Medially conforming knee designs offer an alternative, which aims to minimize paradoxical movement thereby mimicking the native kinematics. They feature asymmetric tibial inserts with a highly congruent medial compartment, alongside a less conforming lateral compartment. With medially stabilized prostheses, a dedicated algorithm different from the other TKA designs is needed during surgery in order to balance the soft tissue. However, little is known which lateral and medial structures must be preserved during surgery with a medially stabilized implant and which structures can be safely released.

Identification of key anatomical structures is crucial for soft tissue balancing; soft tissue balance is of vital importance for good postoperative outcome after TKA [4, 5]. Ligament tightness may cause pain [6], stiffness [7]. and limited range of motion [8], whereas ligament laxity may cause knee instability, a frequent cause of TKA revision [9-11].

To date, there is still no consensus on what structures must be preserved during surgery with a medially stabilized implant and what structures can be safely released. This study therefore analyzed the effect of a progressive selective release of the soft tissues, both laterally and medially, in a knee implanted with a medially stabilized prosthesis.

Aim of the study was to investigate the individual role of medial and lateral soft tissues in medially stabilized replaced knees with different preoperative axes.

## 2 METHODS

Six cadaveric fresh-frozen full leg specimens were used for testing. All specimens had functional ligaments, no history of lower-limb trauma. Three knees had a radiological varus, two a radiological valgus, and one had no radiological deformity. None of the specimens displayed any major signs of osteoarthritis.

For each specimen, the following surgical procedure was conducted. First, the infrared navigation system (iMNS, Medacta International SA, Castel San Pietro, Switzerland) was set up and the anatomic landmarks were acquired. Before any incision was made, the varus/valgus laxity was tested against the range of motion (ROM). Next, a standard medial parapatellar arthrotomy was performed, both meniscus and cruciate ligaments resected and the bony cuts executed using conventional instrumentation and an extension gap first technique, without computer control.

**Fig. (1):**
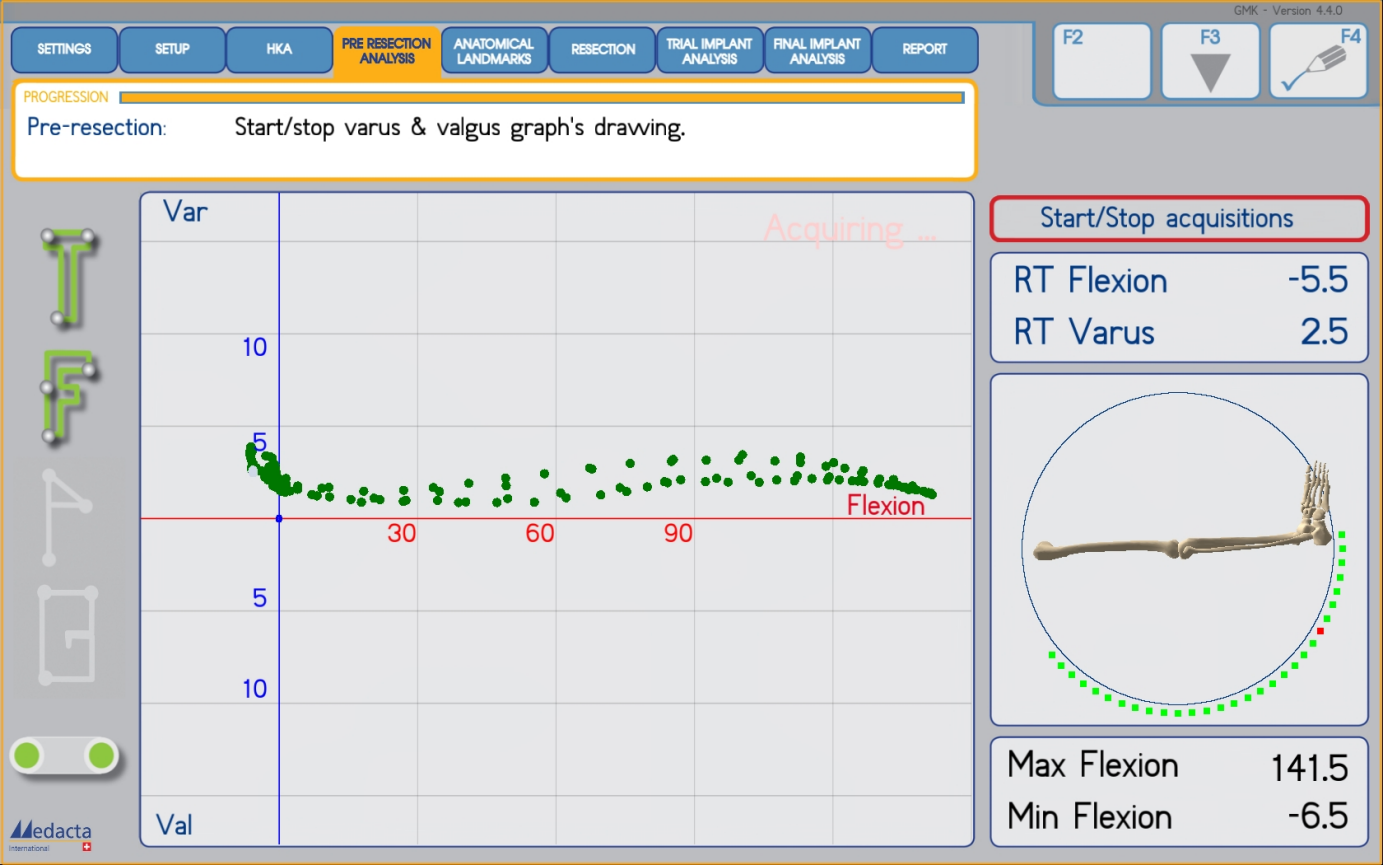
Kinematic pattern of the soft tissue envelope of a varus knee, without applying varus/valgus stress, with the trial components in place from 0° to 120° of flexion.

The trial components (GMK Sphere, Medacta International, Castel San Pietro, Switzerland) were implanted and knee laxity was tested and recorded before any peripheral ligament release (Fig. **1**). Next, progressive release of the soft tissues was performed. The incremental impact of each selective release on knee balance, i.e. varus/valgus laxity of the implanted knee, was subsequently assessed and recoderd in 0°, 30°, 60° and 90° of flexion. Sagittal stability was also manually assessed. The medial and lateral releases were performed on different cadavers so as not to influence the evaluation, i.e. they were not performed on the same specimen.

The sequences of release were always changed to find out the influence of each structure. The released structures were medially: deep medial collateral ligament (dMCL), postero-medial capsule (PMC), partially and complete semimembranosus (SM), posterior and anterior part and complete cut of superficial medial collateral ligament (sMCL).

The released structures were laterally: postero-lateral capsule (PLC), lateral collateral ligament (LCL), popliteus tendon (PT), posterior and anterior fibres of iliotibial band (ITB).

The effect of progressive release on knee balance was measured under navigation control by assessing the angular medial/lateral opening of the joint (degrees) from extension to flexion.

**Fig. (2):**
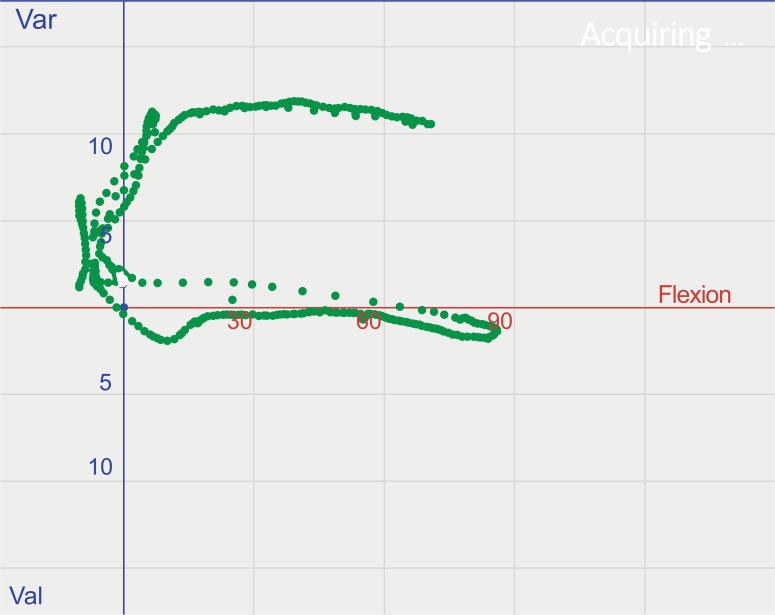
Kinematic pattern of the soft tissue envelope of a varus knee, under application of continuous varus stress and valgus stress moving from extension to flexion and back. Data acquisition was done before any releases.

The opening was induced manually by a single surgeon imposing a varus or valgus stress on the tibia. The varus / valgus stress on the joint was performed as a continuous movement from extension to flexion and back (Fig. **2**).

**Fig. (3):**
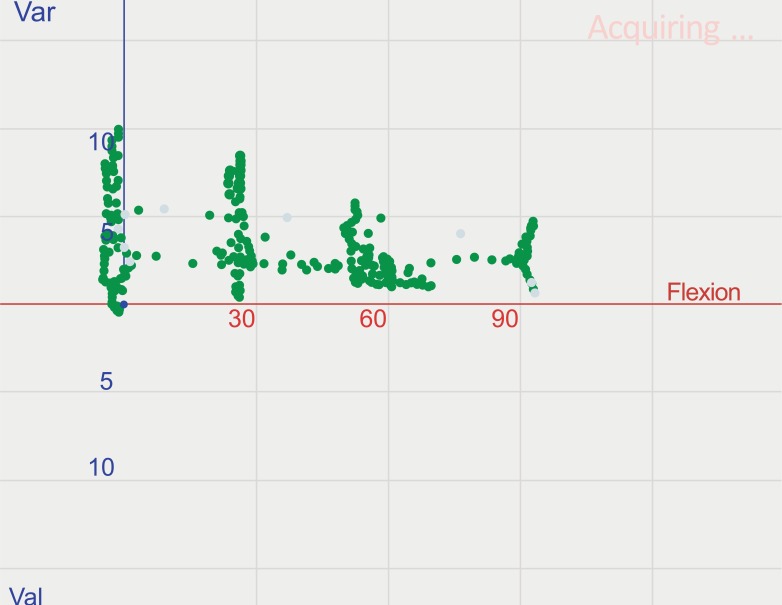
Kinematic pattern of the soft tissue envelope of a varus knee after lateral releases, under application of varus/valgus stress, with the trial components in place, in 0°, 30°, 60° and 90° of flexion.

After performing releases, the opening of the joint was tested in 0°, 30°, 60° and 90° of flexion (Fig. **3**). All stresses were applied by a single surgeon in order to avoid between-surgeon variability.

Analysis of kinematic pattern and medial/lateral laxity of each specimen was conducted at three different intervals: 1: prior to implantation; 2: after the knee was implanted with trial components but before any release of the soft tissues; and 3: progressive release of the soft tissues with the trial implant in place.

## 3 RESULTS

On the medial side, progressive medial release could be performed as long as at least the anterior fibers of the superficial medial collateral ligament (sMCL) remained intact.

With regard to lateral release, in all instances, the progressive release of the lateral soft tissue envelope did not affect sagittal stability, which apparently relies mainly on the medial ball-in-socket design of the implant. Progressive lateral release could be performed as long as the anterior one-third of the iliotibial band (ITB) remained intact.

In the single case where the anterior one-third fibers of the ITB were released before other lateral structures, the effect of instability induced was less evident, and the release did not cause gross instability. However, releasing the anterior fibers of the ITB at the end of the lateral release progression caused complete lateral instability.

In all specimens, it was possible to obtain a knee that was tighter medially and slightly more loose laterally.

## 4 DISCUSSION

**Fig. (4):**
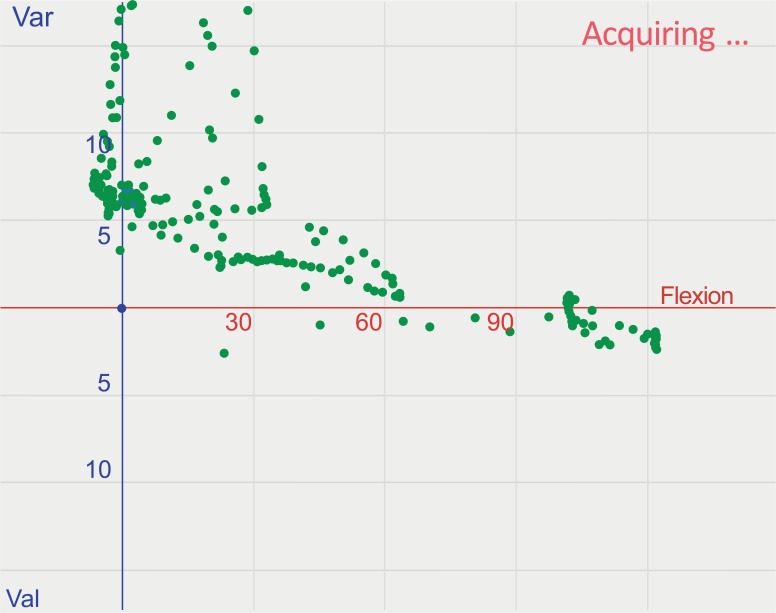
Final release of the anterior fibers of the iliotibial band with laxity tests performed under application of varus/valgus stress, causing complete lateral instability.

The most important finding of the present study is that the anterior-posterior stability of the medially conforming implant remains granted as long as, medially, the anterior fibers of sMCL – and, laterally, the anterior fibers (one-third) of the ITB remain intact (Fig. **4**). It can be inferred that the sagittal stability of the implant relies mostly on the medial ball-in-socket design of the implant.

The anterior fibers of sMCL and the anterior fibers of the ITB are elementary in ensuring joint balance in a knee implanted with a medially stabilized prosthesis. Progressive soft tissue release to balance the knee was feasible, as long as these two structures remain untouched. This study further shows that the sequence of release is relevant. When the anterior ITB is released before the remaining lateral structures, release causes opening along the complete ROM but without creating instability. However, when the anterior ITB was released last, it resulted in gross instability. Hence, the effect on instability of releasing the anterior fibers of the ITB is less evident when this release is done at the beginning rather than at the end of the progressive sequence. This is probably due to the postero-lateral structure still being intact.

Medially conforming knees have shown good mid-term clinical outcome [12, 13] and nearly-normal kinematics, with tibial internal rotation around the medial pivot during active weight-bearing flexion and deep knee flexion, and without the paradoxical anterior translation that is typically seen with conventional cruciate retaining knee designs [14-16].

Although the effect of laxity on functional outcome is a primary concern in TKA, it is difficult to investigate, and a causal relationship has yet to be proven. Preliminary evidence was found by Aunan *et al.* [5] The researchers reported that in a cruciate retaining knee design, functional outcome at one year postoperatively correlated negatively with increasing medial laxity in extension and flexion [5].

Until recently, the objective of TKA has been to obtain rectangular and balanced flexion and extension gaps to ensure stability throughout the range of motion [17-19]. However, recent research has shown that in the native knee, the lateral joint gap is significantly laxer than the medial [20] and that laxity in flexion is larger than in extension [21]. The implication of these differences in laxity is that the tibiofemoral flexion gap in the normal knee is not rectangular and that the symmetric and balanced gap imperative of TKA may lead to overly tight soft tissue restraints relative to those of the native knee. Patients may perceive this as pain, stiffness, and limited flexion [21].

To the best of the authors’ knowledge, this is the first study investigating the effect of progressive soft tissue release on stability of a medially stabilized total knee design. With regard to ligament laxity during TKA, there are few studies that can serve as reference. Based on a radiographic study in healthy subjects, Heesterbeek *et al*. suggested that surgeons should target for valgus laxity in flexion between 0° and 5.5°, and varus laxity in flexion between 0**°** and 7.1**°** [7]. In extension, they recommended valgus laxity between 0.7° and 3.9°, and varus laxity between 0.2° and 5.4°. Bellemans et al recommended a 2–4 mm medial–lateral joint line opening in extension and a 2–6 mm opening in flexion [22]. A recent publication concluded that the amount of force required for a cruciate retaining TKA varied between 48 and 59 N, depending on flexion/extension and compartment. More force was required laterally than medially, and more in flexion than in extension [4]. The latter study concluded that with a balanced gap technique, the lateral compartment must be pre-tensioned less in flexion in order to have a more “natural” laxity of the knee [4].

These aforementioned results do not necessarily apply to medially conforming knees, as these knees have different kinematics and require a dedicated algorithm to manage soft tissue which is different from other design such as cruciate retaining, posterior-stabilized, or mobile bearing. Based on the first author’s surgical experience with a medially stabilized implant, medial releases should be limited since the knee should be tighter medially than laterally. In general, releases are predominantly performed on the lateral side, where a certain laxity is desirable.

In all instances it was possible to achieve a knee which was tighter medially and slightly more loose laterally. Equal medial and lateral laxities can cause lateral soft tissue restraints of the knee to be overly tight, which may be perceived by patients as pain, stiffness, and loss of flexion [21].

Additional studies will be required to further investigate the subject. First, studies are needed to observe the effect of medial and lateral releases on the tibia rotation and natural roll back of the knee implanted with a medially stabilized prosthesis. Second, studies are needed to determine the effect of medial and lateral releases on the intra-articular pressure of the knee implanted with a medially stabilized prosthesis and to quantify the resulting force (Newton) and gap opening (mm). Third, the association between laxity and functional outcome needs to be established. The main problem will be to know which is the ideal laxity or tightness in each individual case.

The study has several limitations. One limitation is that this study was conducted by a single surgeon in cadaveric knees rather than *In vivo*. Releasing a specimen with a contracture will have a different outcome compared with a non-contracture specimen. However, the goal of the study was to assess the importance of the key structures on stability, *i.e*., the anterior fibers of the iliotibial band and anterior fibers of the superficial medial collateral band.

## CONCLUSION

Consequently, the findings are not readily generalizable to *in-vivo* situations. However, the findings are completely in line with the first author’s clinical and surgical experience following this protocol. Second, the sample size was limited. Third, the optimal degree of laxity was not quantitatively measured, rather it was judged more subjectively.

In conclusion, as long as the anterior fibers of sMCL and the anterior fibers of the ITB remain intact, the stability of the medially conforming implant remains granted. The current study found that the sagittal stability of the implant relies mostly on the medial ball-in-socket design of the implant.
